# Supplementary material for: Novel Benzothiazole-Based Ureas as 17β-HSD10 Inhibitors, A Potential Alzheimer’s Disease Treatment
Source: Molecules. 2019 Jul 29;24(15):2757. doi: 10.3390/molecules24152757 (PMC6696238; doi:10.3390/molecules24152757)
Supplement: Supplementary file 1 [file molecules-24-02757-s001.pdf]

Supporting information

Novel benzothiazole-based ureas as 17β-HSD10 inhibitors, a potential Alzheimer's disease treatment

Laura Aitken[1]*, Ondrej Benek[2,3,]*, Brogan E. McKelvie[1], Rebecca E. Hughes[4], Lukas Hroch[2], Monika Schmidt[3], Louise L. Major[5], Lucie Vinklarova[3], Kamil Kuca[3], Terry K. Smith[5], Kamil Musilek[2,3], Frank J. Gunn-Moore[1]

[1]- University of St. Andrews, School of Biology, Medical and Biological Sciences Building, North Haugh, St. Andrews KY16 9TF, United Kingdom

[2]- University Hospital, Biomedical Research Center, Sokolska 581, 500 05 Hradec Kralove, Czech Republic

[3]- University of Hradec Kralove, Faculty of Science, Department of Chemistry, Rokitanskeho 62, 500 03 Hradec Kralove, Czech Republic

[4]- Cancer Research UK Edinburgh Centre, MRC Institute of Genetics and Molecular Medicine, Western General Hospital, University of Edinburgh, Edinburgh, EH4 2XU, United Kingdom

[5]- Biomedical Science Research Complex, University of St. Andrews, North Haugh, St. Andrews KY16 9ST, United Kingdom

*Authors contributed equally to work; corresponding authors

Table of contents

1. Detailed chemical synthesis and intermediate characterization
2. Supporting data from *in vitro* testing
    a. Full primary screen data
    b. IC_{50} graphs
    c. Enzyme kinetics
    d. IC_{50} graphs in cellular model.
3. References

1. Detailed chemical synthesis and intermediate characterization

Procedure A (general conditions):

*N-(6-Methoxybenzo[d]thiazol-2-yl)-1H-imidazole-1-carboxamide (**1**)*

N,N′-Carbonyldiimidazole (12 mmol, 1.95 g) was added to a solution of 6-methoxybenzo[*d*]thiazol-2-amine (10 mmol, 1.80 g) in anhydrous DCM (50 mL) and the reaction was heated at reflux for 20 h.

The reaction mixture was cooled down to 2-8°C, precipitate was collected by suction filtration and washed with cold DCM [1]. The crude product was dried under reduced pressure to yield *N*-(6-methoxybenzo[*d*]thiazol-2-yl)-1*H*-imidazole-1-carboxamide (**1**) as a white solid (2.72 g, 99%) and used in next reaction step without further purification.

Procedure B (general conditions):

*1-Aryl-3-(6-methoxybenzo[d]thiazol-2-yl)ureas (final products **2-6**, **8**, **11**, **28**, **29** and intermediates **7a**, **9a**, **10a**)*

An aromatic amine (1.1 mmol) was added to the suspension of *N*-(6-methoxybenzo[*d*]thiazol-2-yl)-1*H*-imidazole-1-carboxamide (1 mmol) in anhydrous acetonitrile (15 mL) and the reaction was heated at reflux for 20 h [1]. The reaction was cooled to RT, quenched with water (1 M HCl in presence of phenolic group) and the precipitate was collected by suction filtration. The crude product was recrystallised from Et_2O:MeOH and dried to constant weight yielding corresponding 1-aryl-3-(6-methoxybenzobenzo[*d*]thiazol-2-yl)ureas.

*N-Boc-protected urea derivatives (**7a**, **9a**, **10a**)*

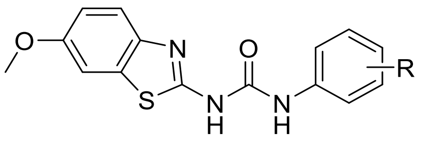

Compound	R	Yield
**7a**	3-NH(Boc), 4-OH	65%
**9a**	4-N(Boc)$_2$	99%
**10a**	3-Cl, 4-N(Boc)$_2$	82%

Compounds **7a**, **9a**, **10a** were used for the next step (*N*-Boc cleavage) without further analysis/ purification.

Procedure C (general conditions):

*N-Boc-cleavage of N-Boc-protected derivatives (**7**, **9**, **10**)*

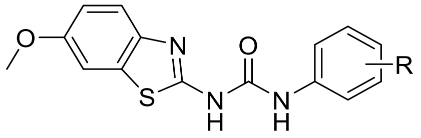

7: R = 3-NH_2, 4-OH
9: R = 4-NH_2
10: R = 3-Cl, 4-NH_2

N-Boc-protected urea derivative (**7a**, **9a**, **10a**, 1 mmol) was stirred for 24 h in 4 M HCl-dioxane solution (10 mL) [2]. Reaction mixture was diluted with Et_2O (50 mL) to achieve complete hydrochloride salt precipitation, which was collected by suction filtration. Solution of 1 M NaOH was added to the mixture of hydrochloride salt in H_2O to achieve desired pH value and mixture was stirred

for next 2 h. Final precipitate was collected by suction filtration, dried and recrystallised (Et_2O:MeCN, 5:1) to yield corresponding urea derivatives (**7**, **9**, **10**).

Procedure D (general conditions):

Palladium on activated carbon catalysed hydrogenation

A nitrobenzene/imine derivative was dissolved in EtOH and palladium on activated carbon (Pd/C, 10%, 0.05 eq) was added. The hydrogen atmosphere was introduced and the mixture was stirred at RT overnight [3]. The mixture was filtered through celite pad and solvent was removed under reduced pressure to yield corresponding final compound.

*4-Amino-2-methylphenol (**12**)*

Beige solid; yield 99%; ^{1}H NMR (500 MHz, DMSO-d_6) δ 8.13 (s, 1H), 6.46 (d, J = 8.3 Hz, 1H), 6.32 (d, J = 2.6 Hz, 1H), 6.22 (dd, J = 8.3, 2.7 Hz, 1H), 4.27 (br s, 2H), 2.00 (s, 3H).

*2-(Tert-butyl)-4-nitrophenol (**13**)*

The solution of 2-(*tert*-butyl)phenol (3 mmol, 0.45 g) in glacial acetic acid (4 mL) was cooled to 5°C and the mixture of nitric acid (3 mmol, 0.19 mL, 70%) in glacial acetic acid (1 mL) was added drop-wise. Reaction mixture was allowed to reach RT and stirred for next 2 h [4]. Then, reaction mixture was poured to cooled H_2O (50 mL) and precipitate was collected by suction filtration. The residue was purified by column chromatography (heptane:EtOAc, 25:1) to yield 2-(*tert*-butyl)-4-nitrophenol as a dark red solid (0.24 g, 41%).

^{1}H NMR (500 MHz, Chloroform-*d*) δ 8.22 (d, J = 2.7 Hz, 1H), 8.01 (dd, J = 8.7, 2.7 Hz, 1H), 6.76 (d, J = 8.7 Hz, 1H), 5.91 (s, 1H), 1.44 (s, 9H).

*4-Amino-2-(tert-butyl)phenol (**14**)*

Ammonium chloride (8 mmol, 0.43 g) and iron powder (20 mmol, 1.12 g) were added to the solution of 2-(*tert*-butyl)-4-nitrophenol (2 mmol, 0.40 g) in MeOH:H_2O (5 mL, 1:1) and the reaction mixture was stirred at RT for next 2 h [5]. The mixture was diluted with THF, filtered through celite pad and solvent was removed under reduced pressure. Crude residue was purified by column chromatography (heptane:EtOAc, 3:1) to yield 4-amino-2-(*tert*-butyl)phenol as a beige solid (0.22 g, 67%).

^{1}H NMR (300 MHz, Chloroform-*d*) δ 6.66 (d, J = 2.6 Hz, 1H), 6.50 (d, J = 8.2 Hz, 1H), 6.43 (dd, J = 8.2, 2.7 Hz, 1H), 1.38 (s, 9H).

*5-Amino-2-hydroxybenzonitrile (**15**)*

5-Amino-2-methoxybenzonitrile (2 mmol, 0.30 g) was suspended in anhydrous DCM (20 mL). Then, aluminium chloride (7 mmol, 0.93 g) was added and the reaction mixture was heated at reflux

overnight. After the reaction was completed (monitored by TLC), water was slowly poured in and the reaction mixture was stirred for another 15 mins. Then the product was extracted to EtOAc (3 × 25 mL), organic layer was washed with brine, dried over anhydrous Na_2SO_4 and solvent was removed under reduced pressure. The crude product was purified by column chromatography ($CHCl_3$:MeOH, 200:1) to obtain 5-amino-2-hydroxybenzonitrile as an orange solid (0.24 g, 88%) and used directly in next synthesis step.

HRMS (ESI) calculated for $C_7H_7N_2O$ $[M+H]^+$ 135.05529, found 135.05533.

Procedure E (general conditions):

N-Boc-protection ***(16-18)***

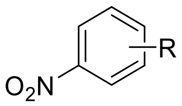

16: R = 3-NH(Boc), 4-OH
17: R = 4-N(Boc)$_2$
18: R = 3-Cl, 4-N(Boc)$_2$

The mixture of an nitro-substituted aromatic amine (3 mmol) and DMAP (0.15 mmol) in anhydrous THF (15 mL) was cooled to 0°C and solution of *tert*-butyl-dicarbonate (6.15 mmol for **57** and **59**; 3.05 mmol for **58**) in anhydrous THF (5 mL) was added drop-wise. Reaction mixture was allowed to reach RT, stirred overnight and heated at 65°C for 2 h to complete the reaction [2]. Solvent was removed under reduced pressure, residue was diluted with EtOAc (75 mL), washed with citric acid (15%, 2 × 15 mL), water (15 mL) and brine (15 mL). Organic layer was dried over anhydrous Na_2SO_4, solvent removed under reduced pressure and crude residue was used without further purification.

Reduction of N-Boc-protected nitro-derivatives ***(19-21)***

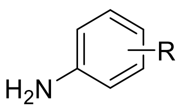

19: R = 3-NH(Boc), 4-OH
20: R = 4-N(Boc)$_2$
21: R = 3-Cl, 4-N(Boc)$_2$

Procedure **D** (Pd/C catalysed hydrogenation) was used to obtain corresponding *N*-Boc-protected aniline derivative and crude product was used in next step without further purification.

(4-Amino-2-chlorophenyl)methanol ***(22)***

Lithium aluminium hydride (9 mmol, 0.34 g) was suspended in anhydrous THF (5 mL) at -5°C and suspension was stirred 1 h at -5°C under nitrogen atmosphere. Solution of 4-amino-2-chlorobenzoic acid (3 mmol, 0.51 g) in anhydrous THF (3 mL) was added dropwise over the 30 min time period [6]. The reaction mixture was allowed to reach RT and stirred overnight. The reaction was quenched with H_2O, alkalised with saturated $NaHCO_3$ solution and extracted to EtOAc (3 × 25 mL). The organic layers were combined, dried over anhydrous Na_2SO_4 and solvent was removed under reduced pressure. The residue

was purified by column chromatography ($CHCl_3$:MeOH, 500:1) to yield (4-amino-2-chlorophenyl)methanol as a beige solid (0.46 g, 98%).

^{1}H NMR (500 MHz, DMSO-d_6) δ 7.11 (d, *J* = 8.2 Hz, 1H), 6.57 (d, *J* = 2.2 Hz, 1H), 6.49 (dd, *J* = 8.2, 2.2 Hz, 1H), 5.26 (s, 2H), 4.95 (t, *J* = 5.5 Hz, 1H), 4.37 (d, *J* = 5.5 Hz, 2H).

*2-chloro-4-((6-chlorobenzo[d]thiazol-2-yl)amino)phenol (**23**)*

2,6-dichlorobenzo[*d*]thiazole (1 eq.) and 4-amino-2-chlorophenol (1.05 eq.) were dissolved in *N*-methyl-2-pyrrolidone (NMP; 3mL/mmol) and the reaction mixture was stirred at 160°C overnight. After the reaction was completed (monitored by TLC), 1M aq. HCl was added to the reaction mixture and the water layer was extracted with EtOAc. The water layer was adjusted to pH=7 and extracted with EtOAc. The organic layer was evaporated and the crude residue was purified using column chromatography to obtain the product in 30% yield.

*N-(3-chloro-4-hydroxyphenyl)-6-methoxybenzo[d]thiazole-2-carboxamide (**24**)*

6-methoxybenzo[*d*]thiazole-2-carboxylic acid (1 eq.) was dissolved in anhydrous DMF (7 mL/mmol), CDI (1.1 eq.) was added and the reaction mixture was stirred at RT for 1 h. Then 4-amino-2-chlorophenol (1.1 eq.) was added and the reaction was stirred at RT overnight. After the reaction was completed (monitored by TLC), 1M aq. HCl was added to the reaction mixture, resulting precipitate was collected by filtration and recryctallized from MeCN to obtain *N*-(3-chloro-4-hydroxyphenyl)-6-methoxybenzo[*d*]thiazole-2-carboxamide in 58% yield.

*3-chloro-4-methoxy-N-(6-methoxybenzo[d]thiazol-2-yl)benzamide (**25**)*

3-chloro-4-methoxybenzoic acid (1 eq.) was dissolved in anhydrous DMF (7 mL/mmol), CDI (1.1 eq.) was added and the reaction mixture was stirred at RT for 2 h. Then 6-methoxybenzo[*d*]thiazol-2-amine (1.1 eq.) was added and the reaction was stirred at RT overnight. After the reaction was completed (monitored by TLC), 1M aq. HCl was added to the reaction mixture and the resulting precipitate was collected by filtration and dried. The crude product was purified by column chromatography to obtain 3-chloro-4-methoxy-*N*-(6-methoxybenzo[*d*]thiazol-2-yl)benzamide in 34% yield.

Procedure F (general conditions):

O-demethylation using aluminium trichloride

Corresponding phenylmethylether (1 eq.) was dissolved/dispersed in anhydrous toluene (12 mL/mmol), aluminium chloride (3.5 eq.) was added and the reaction mixture was stirred at reflux overnight. After the reaction was completed (monitored by TLC), water was slowly poured in and the

reaction mixture was stirred for another 15 mins. Then the product was extracted to EtOAc, organic layer was washed with brine, dried with anhydrous Na_2SO_4 and evaporated. The crude product was either further purified (described separately for respective compounds) or used as such.

*3-Chloro-4-hydroxy-N-(6-methoxybenzo[d]thiazol-2-yl)benzamide (**26**)*

3-chloro-4-hydroxy-*N*-(6-methoxybenzo[*d*]thiazol-2-yl)benzamide was prepared from 3-chloro-4-methoxy-*N*-(6-methoxybenzo[*d*]thiazol-2-yl)benzamide according general procedure **F** using $AlCl_3$ described above. The crude product was purified by column chromatography to obtain compound **26** in 69% yield.

*4-(aminomethyl)-2-chlorophenol (**27**)*

4-(aminomethyl)-2-chlorophenol was prepared from (3-chloro-4-methoxyphenyl)methanaminium chloride by demethylation according to the general procedure **F**. After the reaction was completed (monitored by TLC), water was slowly poured in and the reaction mixture was stirred for another 15 mins. The organic layer was removed and the water layer was filtered and the filtrate evaporated. The solid residue was dispersed in acetone using the ultrasound bath and filtered. Filtrate was evaporated to give 4-(aminomethyl)-2-chlorophenol (92%), which was used for the next step without further purification.

*1-(3-chloro-4-hydroxybenzyl)-3-(6-methoxybenzo[d]thiazol-2-yl)ureas (**28**, **29**)*

Final product 28 and 29 with prolonged urea linker were prepared from intermediate **1** in reaction with corresponding benzylamine according to general procedure **B**.

Procedure G (general conditions):

*Schiff bases synthesis (**30-33**)*

Equimolar amounts of amine and aldehyde were dissolved in toluene (10 mL) and heated at reflux for 48 h. Solvent was removed under reduced pressure and the crude residue was recrystallized (PE:Et_2O) to yield corresponding product as an yellow solid.

*(E)-1-(4-Fluorophenyl)-N-(6-methoxybenzo[d]thiazol-2-yl)methanimine (**30**)*

Yellow solid; yield 88%; ^{1}H NMR (500 MHz, DMSO-d_6) δ 9.15 (s, 1H), 8.20 – 8.12 (m, 2H), 7.83 (d, *J* = 8.9 Hz, 1H), 7.67 (d, *J* = 2.4 Hz, 1H), 7.47 – 7.40 (m, 2H), 7.12 (dd, *J* = 8.9, 2.6 Hz, 1H), 3.84 (s, 3H).

*(E)-4-(((6-Methoxybenzo[d]thiazol-2-yl)imino)methyl)phenol (**31**)*

Yellow solid; yield 93%; ^{1}H NMR (500 MHz, DMSO-d_6) δ 10.55 (s, 1H), 8.97 (s, 1H), 7.93 (d, *J* = 8.7 Hz, 2H), 7.78 (d, *J* = 8.9 Hz, 1H), 7.62 (d, *J* = 2.6 Hz, 1H), 7.08 (dd, *J* = 8.9, 2.6 Hz, 1H), 6.94 (d, *J* = 8.6 Hz, 2H), 3.83 (s, 3H).

*Methyl (E)-2-hydroxy-5-(((6-methoxybenzo[d]thiazol-2-yl)imino)methyl)benzoate (**32**)*

Yellow solid; yield 80%; ^{1}H NMR (500 MHz, DMSO-d_6) δ 11.14 (s, 1H), 9.08 (s, 1H), 8.50 (d, *J* = 2.2 Hz, 1H), 8.19 (dd, *J* = 8.7, 2.2 Hz, 1H), 7.81 (d, *J* = 8.9 Hz, 1H), 7.65 (d, *J* = 2.6 Hz, 1H), 7.17 (d, *J* = 8.6 Hz, 1H), 7.10 (dd, *J* = 8.9, 2.7 Hz, 1H), 3.93 (s, 3H), 3.84 (s, 3H).

*(E)-2-Chloro-4-(((6-methoxybenzo[d]thiazol-2-yl)imino)methyl)phenol (**33**)*

Yellow solid; yield 84%; m.p. 260.5–261.5 °C; ^{1}H NMR (500 MHz, DMSO-d_6) δ 11.34 (s, 1H), 8.99 (s, 1H), 8.07 (d, *J* = 2.1 Hz, 1H), 7.90 (dd, *J* = 8.5, 2.1 Hz, 1H), 7.80 (d, *J* = 8.8 Hz, 1H), 7.64 (d, *J* = 2.6 Hz, 1H), 7.14 (d, *J* = 8.5 Hz, 1H), 7.10 (dd, *J* = 8.9, 2.6 Hz, 1H), 3.84 (s, 3H). ^{13}C NMR (126 MHz, DMSO-d_6) δ 168.94, 164.36, 157.82, 157.18, 145.55, 135.28, 131.85, 130.29, 127.05, 123.08, 120.64, 117.06, 115.64, 105.09, 55.70. HRMS (ESI) calcd for $C_{15}H_{12}ClN_2O_2S$ $[M+H]^+$ 319.03025, found 319.0302.

Procedure H (general conditions):

*Dimethyl (aryl((6-methoxybenzo[d]thiazol-2-yl)amino)methyl) phosphonates (**34-37**)*

A Schiff base (**30-33**, 1 mmol) was suspended in THF (5 mL) followed by immediate addition of dimethyl phosphite (1.1 mmol) and 1,1,3,3-tetramethylguanidine (1.1 mmol) [7]. The mixture was heated at 65°C for next 12 h, then solvent was removed under reduced pressure and residue was purified by column chromatography ($CHCl_3$:MeOH). The crude product was recrystallized (PE:Et_2O) to yield corresponding phosphonate.

*N-methylbenzo[d]thiazol-2-amine (**38**)*

2-iodoaniline (1 eq.), methylisothiocyanate (1 eq.), tetrabutylammonium bromide (1 eq.) and copper (I) chloride (0.01 eq.) were dissolved in DMSO (7 mL/mmol) and the reaction mixture was stirred at 80°C overnight. After the reaction was completed (monitored by TLC), water was added to the reaction mixture and the product was extracted to Et_2O. Organic layer was washed with brine, dried using anhydrous Na_2SO_4 and concentrated under reduced pressure. The crude product was purified by column chromatography to obtain *N*-methylbenzo[*d*]thiazol-2-amine in 40% yield.

^{1}H NMR (500 MHz, DMSO-d_6): δ (ppm) 7.91 (d, *J* = 4.4 Hz, 1H), 7.65 (dd, *J* = 7.8, 0.9 Hz, 1H), 7.39 (dd, *J* = 8.0, 0.6 Hz, 1H), 7.21 (ddd, *J* = 8.1, 7.4, 1.3 Hz, 1H), 7.05 – 6.96 (m, 1H), 2.93 (d, *J* = 4.7 Hz, 3H).

*2-chloro-4-isocyanato-1-methoxybenzene (**39**)*

Triphosgene (1 eq.) was dissolved in anh. DCM (3.5 mL/mmol) at 0 °C and 3-chloro-4-methoxyaniline (1 eq.), dissolved in anh. DCM, was added dropwise. Consequently, Et_3N (0.3

mL/mmol) was added dropwise and the reaction mixture was stirred at reflux for 1 h. Then the reaction was cooled to the room temperature, Et_2O was added, the mixture was filtered and washed with Et_2O. The filtrate was concentrated under reduced pressure to obtain the crude product (100%), which was used without further purification for the next step.

*1-(benzo[d]thiazol-2-yl)-3-(3-chloro-4-methoxyphenyl)-1-methylurea (**40**)*

N-methylbenzo[*d*]thiazol-2-amine (1 eq.) was dissolved in anh. THF (6 mL/mmol), 2-chloro-4-isocyanato-1-methoxybenzene (0.95 eq.), dissolved in anh. THF, was added dropwise and the reaction mixture was stirred at reflux overnight. After the reaction was completed (monitored by TLC), 1M aq. HCl (1 mL/mmol) was added dropwise and the reaction mixture was evaporated to dryness under reduced pressure. The crude residue was dispersed in MeOH in the ultrasound bath, filtered and washed with MeOH to give the product in 67% yield.

^{1}H NMR (500 MHz, DMSO-d_6): δ (ppm) 9.54 (s, 1H), 7.91 (dd, *J* = 7.8, 0.6 Hz, 1H), 7.75 (d, *J* = 8.0 Hz, 1H), 7.69 (d, *J* = 2.6 Hz, 1H), 7.49 (dd, *J* = 8.9, 2.6 Hz, 1H), 7.44 – 7.37 (m, 1H), 7.30 – 7.23 (m, 1H), 7.16 (d, *J* = 9.0 Hz, 1H), 3.85 (s, 3H), 3.76 (s, 3H).

*1-(benzo[d]thiazol-2-yl)-3-(3-chloro-4-hydroxyphenyl)-1-methylurea (**41**)*

Compound **41** was prepared from 1-(benzo[*d*]thiazol-2-yl)-3-(3-chloro-4-methoxyphenyl)-1-methylurea according to the general demethylation procedure **F**. The crude product was purified by column chromatography to obtain compound **41** in 83% yield.

*3-chloro-4-methoxy-N-methylaniline (**42**)*

3-chloro-4-methoxyaniline (1 eq.) was dissolved in anh. THF (4 mL/mmol), sodium hydride (1.5 eq.) was added at 0°C and the reaction mixture was stirred for 30 mins. Then methyl iodide (1.1 eq.) was added and the reaction was allowed to warm up to rt and stirred overnight. After the reaction was completed (monitored by TLC), water was slowly added into the reaction and product was extracted to EtOAc. The organic layer was washed with brine, dried using anhydrous Na_2SO_4 and concentrated under reduced pressure. The crude product was purified by column chromatography to obtain 3-chloro-4-methoxy-*N*-methylaniline in 28% yield.

^{1}H NMR (500 MHz, $CDCl_3$): δ (ppm) 6.82 (d, *J* = 8.8 Hz, 1H), 6.68 (d, *J* = 2.7 Hz, 1H), 6.50 (dd, *J* = 8.8, 2.7 Hz, 1H), 3.82 (s, 3H), 2.79 (s, 3H).

*2-chloro-4-(methylamino)phenol (**43**)*

2-chloro-4-(methylamino)phenol was prepared from 3-chloro-4-methoxy-*N*-methylaniline according to the general demethylation procedure **F**. The crude product was purified by column chromatography to obtain the product in 74% yield.

^{1}H NMR (500 MHz, $CDCl_3$): δ (ppm) 6.86 (d, *J* = 8.7 Hz, 1H), 6.59 (d, *J* = 2.8 Hz, 1H), 6.48 (dd, *J* = 8.7, 2.8 Hz, 1H), 2.78 (s, 3H).

*N-(benzo[d]thiazol-2-yl)-1H-imidazole-1-carboxamide (**44**)*

N-(benzo[d]thiazol-2-yl)-1H-imidazole-1-carboxamide was prepared from benzo[*d*]thiazol-2-amine and CDI according to the general procedure **A** in 88% yield.

*3-(benzo[d]thiazol-2-yl)-1-(3-chloro-4-hydroxyphenyl)-1-methylureas (**45**, **46**)*

Mono-methylated urea derivatives **45** and **46** were prepared from intermediates **44** resp. **1** in reaction with 2-chloro-4-(methylamino)phenol (**43**) according to the general procedure **B**.

*3-(benzo[d]thiazol-2-yl)-1-(3-chloro-4-methoxyphenyl)-1-methylurea (**47**)*

3-(benzo[*d*]thiazol-2-yl)-1-(3-chloro-4-methoxyphenyl)-1-methylurea was prepared from carboxamide **44** and 3-chloro-4-methoxy-*N*-methylaniline according to the general procedure **B** in 91% yield.

*1-(benzo[d]thiazol-2-yl)-3-(3-chloro-4-methoxyphenyl)-1,3-dimethylurea (**48**)*

3-(benzo[*d*]thiazol-2-yl)-1-(3-chloro-4-methoxyphenyl)-1-methylurea (1 eq.) was dissolved in anh. DMF (10 mL/mmol), sodium hydride (1.2 eq.) was added at 0°C and the reaction mixture was stirred for 30 mins. Then methyl iodide (2.2 eq.) was added and the reaction was allowed to warm up to rt and stirred overnight. After the reaction was completed (monitored by TLC), water was slowly added and product was extracted to EtOAc. The organic layer was washed with brine, dried using anhydrous Na_2SO_4 and concentrated under reduced pressure. The crude product was recrystallized from MeOH to obtain the product in 77% yield.

^{1}H NMR (500 MHz, DMSO-d_6): δ (ppm) 7.76 (d, *J* = 7.7 Hz, 1H), 7.49 (d, *J* = 2.6 Hz, 1H), 7.48 – 7.39 (m, 2H), 7.31 (dd, *J* = 8.8, 2.5 Hz, 1H), 7.25 (t, *J* = 8.0 Hz, 1H), 7.13 (d, *J* = 8.9 Hz, 1H), 3.87 (s, 3H).

*1-(benzo[d]thiazol-2-yl)-3-(3-chloro-4-hydroxyphenyl)-1,3-dimethylurea (**49**)*

Final product **49** was prepared from 1-(benzo[*d*]thiazol-2-yl)-3-(3-chloro-4-methoxyphenyl)-1,3-dimethylurea (**48**) according to the general demethylation procedure **F**. The crude product was purified by column chromatography to obtain compound **49** in 65% yield.

Procedure I (general conditions):

*Synthesis of 6-substituted benzo[d]thiazol-2-amines (**50**, **51**, **53**)*

Corresponding para-substituted aniline (1 eq.) and KSCN (4 eq.) were dissolved in acetic acid (4 mL/mmol) and stirred at rt for 20 mins. Then the reaction mixture was cooled to 10 °C and bromine (2 eq.) dissolved in small amount of acetic acid was added dropwise. Afterwards the reaction mixture was left to warm up to RT and stirred overnight. After the reaction was completed (monitored by TLC), reaction mixture was added dropwise into the sat. aq. NH_3 solution (15 mL/mmol) while cooling in an ice bath. The product was extracted to EtOAc and the organic layer was washed with $Na_2S_2O_3$, sat. aq. $NaHCO_3$ and brine, dried using anhydrous Na_2SO_4 and concentrated under reduced pressure. The crude product was recrystallized from diethylether.

*6-((trifluoromethyl)thio)benzo[d]thiazol-2-amine (**50**)*

Yield 23%

*6-((trifluoromethyl)sulfonyl)benzo[d]thiazol-2-amine (**51**)*

Yield 26%; ^{1}H NMR (500 MHz, DMSO-d_6): δ (ppm) 8.53 (d, J = 2.0 Hz, 1H), 8.40 (s, 2H), 7.84 (dd, J = 8.6, 2.1 Hz, 1H), 7.58 (d, J = 8.6 Hz, 1H).

*6-thiocyanatobenzo[d]thiazol-2-amine (**53**)*

Yield 32%

*6-(tert-butyl)benzo[d]thiazol-2-amine (**52**)*

4-(tert-butyl)aniline (1 eq.) and KSCN (7 eq.) were dissolved in mixture of DMSO/H_2O (9:1; 10 mL/mmol). Tetramethylammonium dichloroiodate (3 eq.) was added and the reaction mixture stirred at RT for 5 mins and then at 70 °C overnight [8]. After the reaction was completed (monitored by TLC), water was poured into the reaction and the product extracted to EtOAc. The organic layer was washed with $Na_2S_2O_3$, sat. aq. $NaHCO_3$ and brine, dried using anhydrous Na_2SO_4 and concentrated under reduced pressure. The crude product was used for the next step without further purification (98%).

^{1}H NMR (500 MHz, DMSO-d_6): δ (ppm) 7.65 (m, 1H), 7.33 (br s, 2H), 7.25 – 7.22 (m, 2H), 1.28 (s, 9H).

*6-substituted N-(benzo[d]thiazol-2-yl)-1H-imidazole-1-carboxamides (**54-60**)*

Carboxamides **54-60** were prepared from corresponding 6-substituted benzo[*d*]thiazol-2-amines and CDI according to the general procedure **A**.

Compound	R	Yield
**54**	*i*-propyl	77%
**55**	*t*-butyl	66%
**56**	-OEt	76%
**57**	-SCF_3	70%
**58**	-SCN	67%
**59**	-SO_2Me	85%
**60**	-SO_2CF_3	40%

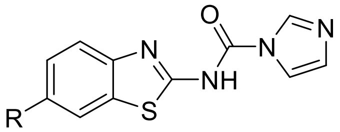

*6-substituted 1-(benzo[d]thiazol-2-yl)-3-(3-chloro-4-hydroxyphenyl)ureas (**61-67**)*

Final products **61-67** were prepared according to the general procedure **B**.

*N-(thiazol-2-yl)-1H-imidazole-1-carboxamide derivatives (**68-70**)*

Intermediates **68-70** were prepared from corresponding thiazol-2-amines derivatives and CDI according to the general procedure **A**.

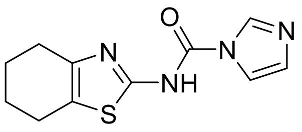

68 (78%)

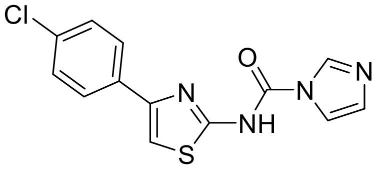

69 (65%)

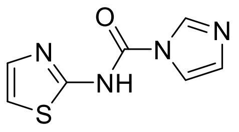

70 (74%)

*1-(3-chloro-4-hydroxyphenyl)-3-(thiazol-2-yl)urea derivatives (***71***,* **73***,* **74***)*

Final products **71**, **73**, **74** were prepared according to the general procedure **B**.

*1-(3-chloro-4-hydroxyphenyl)-3-(2,3-dihydro-1H-inden-2-yl)urea (**72**)*

2,3-dihydro-1*H*-inden-2-amine (1 eq.) was dissolved in DMF (3 mL/mmol), CDI (1.1 eq.) was added and the reaction mixture was stirred at 50 °C for 6 h. Then 4-amino-2-chlorophenol (1.1 eq.) was added and the reaction was stirred at 70 °C overnight. After the reaction was completed (monitored by TLC), 1M aq. HCl was added to the reaction mixture and resulting precipitate was collected by filtration. The crude product was purified using column chromatography to obtain *N*-(3-chloro-4-hydroxyphenyl)-6-methoxybenzo[*d*]thiazole-2-carboxamide in 21% yield.

*1-(3-chloro-4-hydroxyphenyl)-3-(4-methoxyphenethyl)urea (**75**)*

2-(4-methoxyphenyl)ethan-1-amine (1 eq.) was dissolved in DMF (7 mL/mmol), CDI (1.1 eq.) was added and the reaction mixture was stirred at 45 °C for 6 h. Then 4-amino-2-chlorophenol (1.1 eq.) was added and the reaction was stirred at 70 °C overnight. After the reaction was completed (monitored by TLC), 1M aq. HCl was added to the reaction mixture and resulting precipitate was collected by filtration. The crude product was purified using column chromatography to obtain 1-(3-chloro-4-hydroxyphenyl)-3-(4-methoxyphenethyl)urea in 21% yield.

*1-(benzo[d]thiazol-6-yl)-3-(3-chloro-4-hydroxyphenyl)urea (**76**)*

Benzo[*d*]thiazol-6-amine was dissolved in a mixture of DCM and DMF (6:1; 12 mL/mmol). CDI (1.1 eq.) was added and the reaction mixture was stirred at reflux overnight. The resulting precipitate was collected by filtration and removed. Filtrate was concentrated, dissolved in MeCN (10 mL/mmol), 4-amino-2-chlorophenol (1.1 eq.) was added and the reaction was stirred at reflux overnight. After the reaction was completed (monitored by TLC), 1M aq. HCl was added to the reaction mixture and resulting precipitate was collected by filtration. The crude product was purified using column chromatography to obtain 1-(benzo[*d*]thiazol-6-yl)-3-(3-chloro-4-hydroxyphenyl)urea in 25% yield.

*1,3-bis(3-chloro-4-methoxyphenyl)urea (**77**)*

3-chloro-4-methoxyaniline (2 eq.) was dissolved in DMF (3 mL/mmol), CDI (1 eq.) was added and the reaction mixture was stirred at 60°C overnight. After the reaction was completed (monitored by TLC), 1M aq. HCl was added to the reaction mixture and resulting precipitate was collected by filtration, washed with water and dried to give 1,3-bis(3-chloro-4-methoxyphenyl)urea in 90% yield.

^{1}H NMR (300 MHz, DMSO-d_6): δ (ppm) 8.62 (br s, 2H), 7.64 (d, J = 2.2 Hz, 2H), 7.25 (dd, J = 8.9, 2.2 Hz, 2H), 7.07 (d, J = 8.9 Hz, 2H), 3.80 (s, 6H).

*1,3-bis(3-chloro-4-hydroxyphenyl)urea (**78**)*

1,3-bis(3-chloro-4-hydroxyphenyl)urea was prepared from 1,3-bis(3-chloro-4-methoxyphenyl)urea according to the General demethylation procedure using $AlCl_3$ described above (see 2-aminobenzo[*d*]thiazol-6-ol synthesis). Toluene reflux

The crude product was recrystallized from Et_2O to obtain compound **78** in 94% yield.

2. Supporting data from *in vitro* testing

2.a. Full primary screen data

Table S1: Full primary screen compound *in vitro* evaluation (Relative remaining activity is displayed in percentage of six independent measurements ± SEM).

Compound ID	17β-HSD10 activity in the presence of compound at 25 µM (Primary Screen)	Compound ID	17β-HSD10 activity in the presence of compound at 25 µM (Primary Screen)

**control**	100 ± 1.68	**45**	77.33 ± 1.43
**2**	52.24 ± 3.67	**46**	97.85 ± 1.32
**3**	41.01 ± 0.45	**49**	104.80 ± 2.21
**4**	96.50 ± 2.03	**61**	36.48 ± 0.53
**5**	14.34 ± 1.95	**62**	33.96 ± 2.44
**6**	9.82 ± 2.19	**63**	28.73 ± 0.38
**7**	82.49 ± 2.39	**64**	50.54 ± 1.25
**8**	63.33 ± 3.42	**65**	37.83 ± 0.35
**9**	105.20 ± 3.17	**66**	21.62 ± 0.21
**10**	117.00 ± 3.94	**67**	29.10 ± 0.32
**11**	109.80 ± 3.80	**71**	55.75 ± 1.54
**23**	120.40 ± 13.47	**72**	79.17 ± 2.56
**24**	54.18 ± 4.40	**73**	102.30 ± 3.65
**26**	105.40 ± 4.99	**74**	74.50 ± 0.63
**28**	101.30 ± 4.60	**75**	73.34 ± 4.33
**29**	63.55 ± 2.58	**76**	43.80 ± 0.77
**34**	95.40 ± 1.16	**78**	35.24 ± 2.31
**35**	96.58 ± 1.35	**K690***	38.8 ± 0.5
**36**	95.87 ± 2.09	**K691***	38.6 ± 0.7
**37**	97.83 ± 0.88	**K822***	17.3 ± 0.8
**41**	106.10 ± 20.93	**K824***	20.3 ± 1.1

*Authors previously published most potent enzymatic data from Hroch *et al.* 2016 (Design, synthesis and in vitro evaluation of benzothiazole-based ureas as potential ABAD/17β-HSD10 modulators for Alzheimer's disease treatment).

2.b. IC_{50} graphs

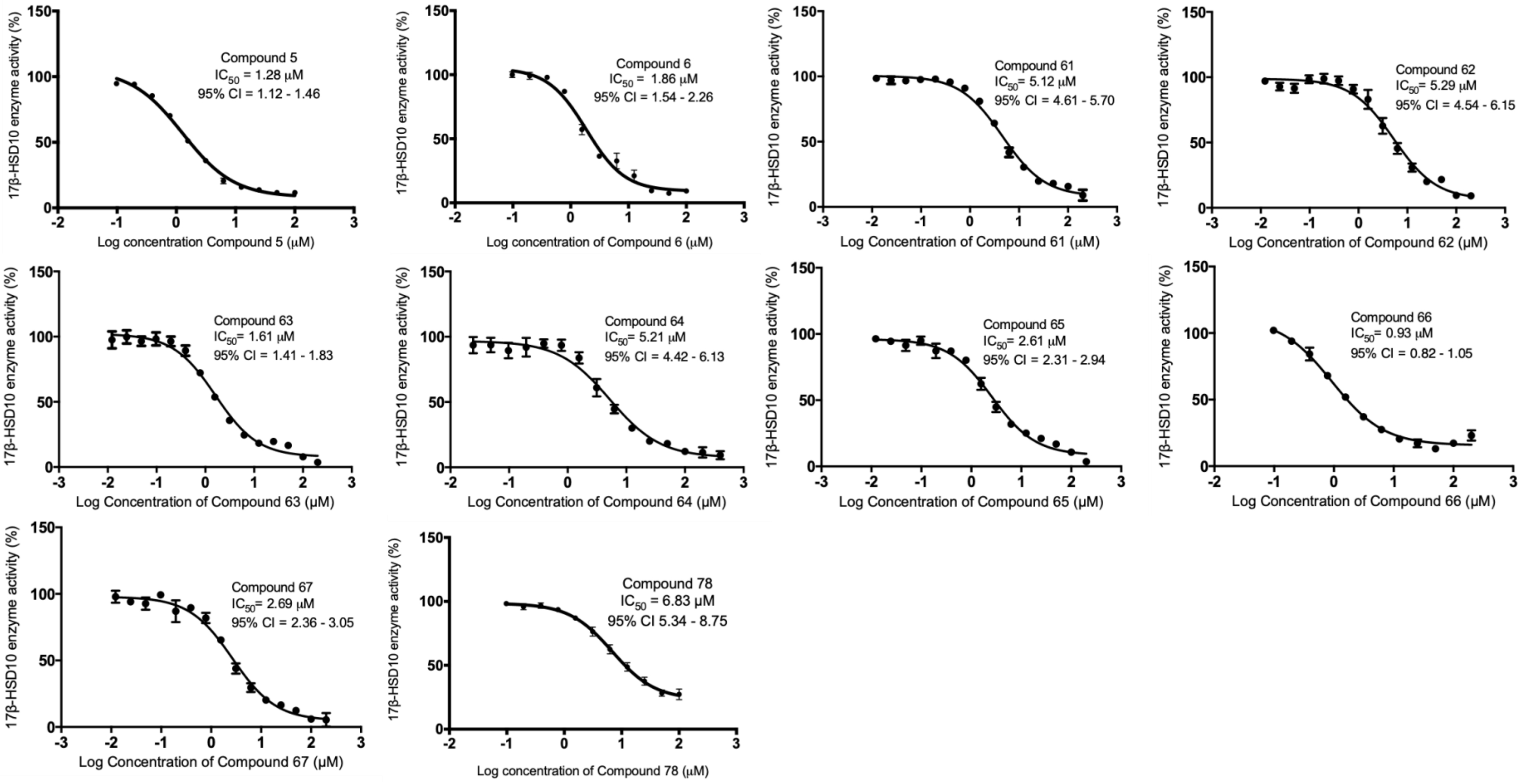

Figure [S1]: IC_{50} Graphs for compounds **5**, **6**, **61**, **62**, **63**, **64**, **65**, **66**, **67** and **78**.

2.c. Enzyme kinetics

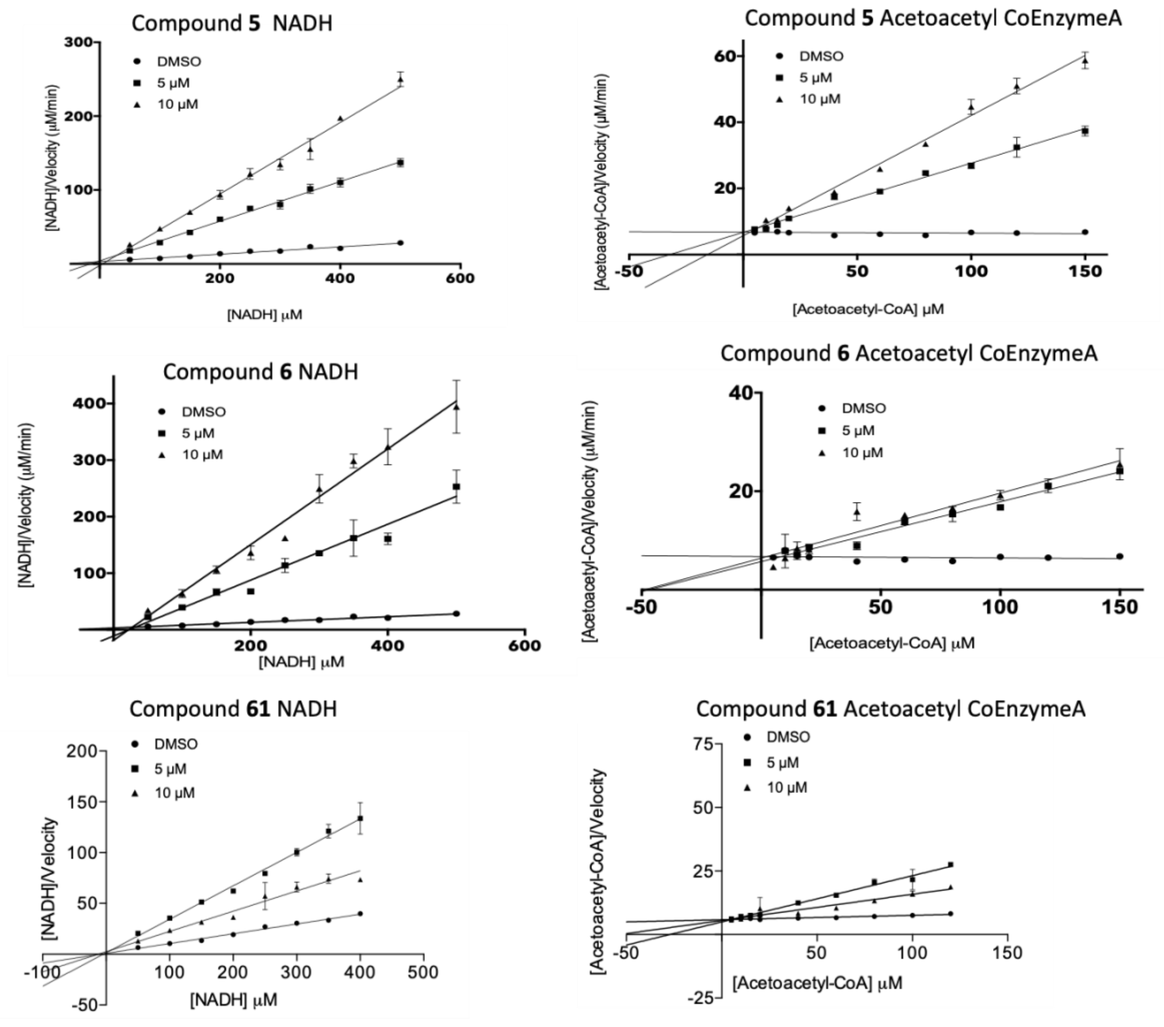

Figure [S2]: Hanes-Woolf kinetic plots for compounds **5**, **6** and **61**. Each compound's mode of inhibition with respect to both Acetoacetyl CoEnzymeA and NADH determined from Hanes-Woolf calculations. The mean values shown with n=3 ± St Dev.

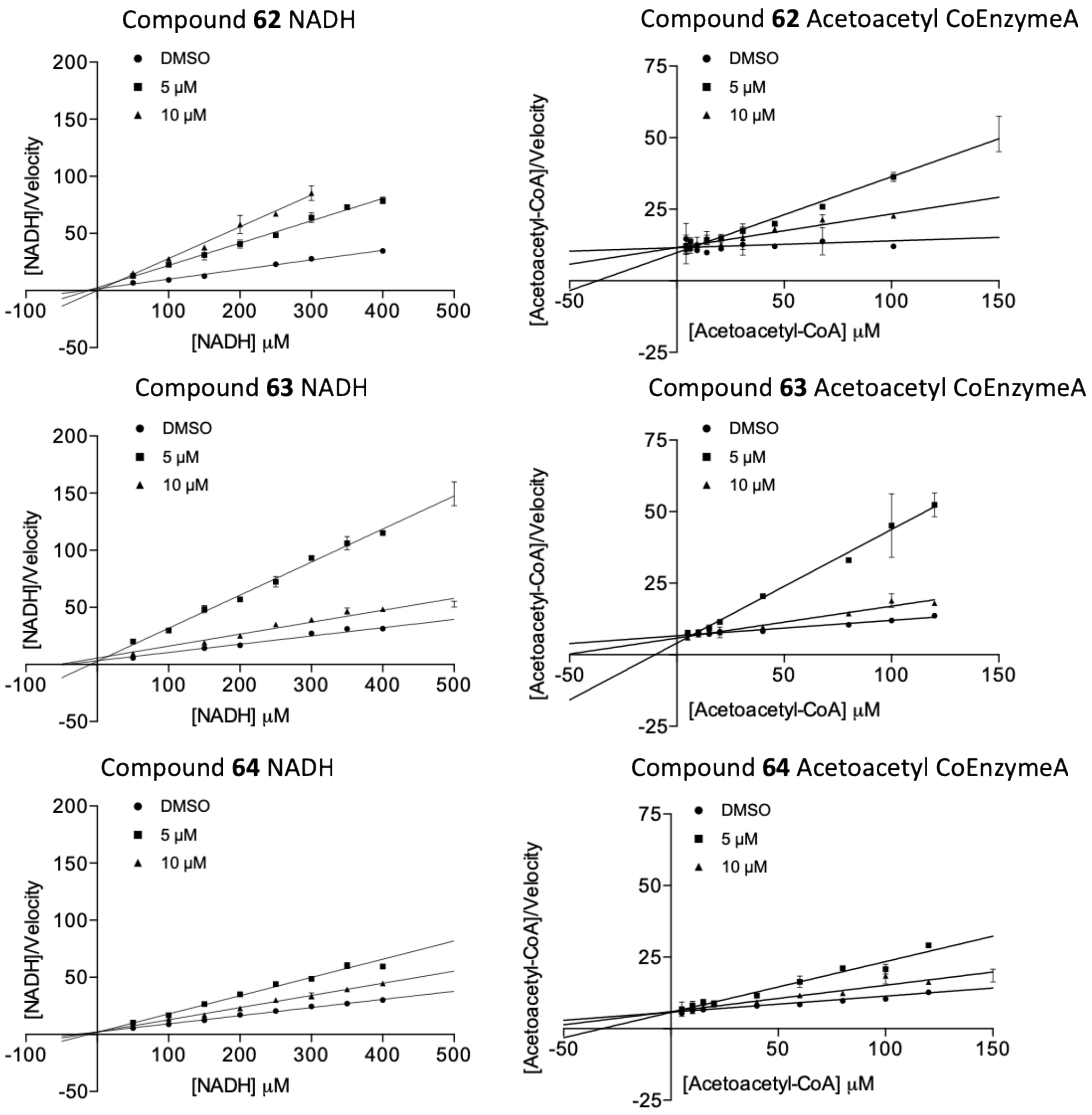

Figure [S3]: Hanes-Woolf kinetic plots for compounds **62**, **63** and **64**. Each compound's mode of inhibition with respect to both Acetoacetyl CoEnzymeA and NADH determined from Hanes-Woolf calculations. The mean values shown with n=3 ± St Dev.

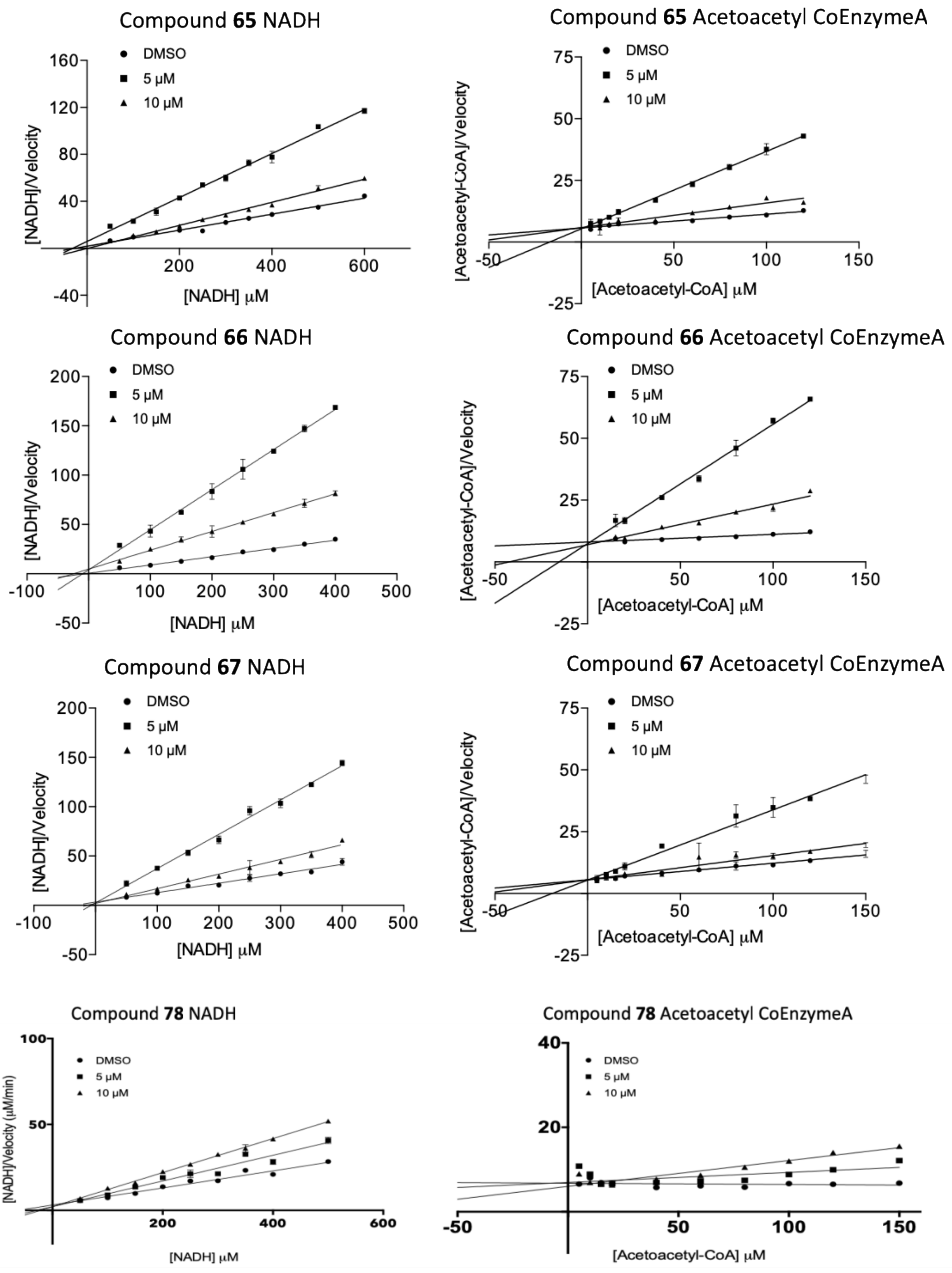

Figure [S4]: Hanes-Woolf kinetic plots for compounds **65**, **66**, **67** and **61**. Each compound's mode of inhibition with respect to both Acetoacetyl CoEnzymeA and NADH determined from Hanes-Woolf calculations. The mean values shown with n=3 ± St Dev.

2.d. Cellular IC_{50} determination

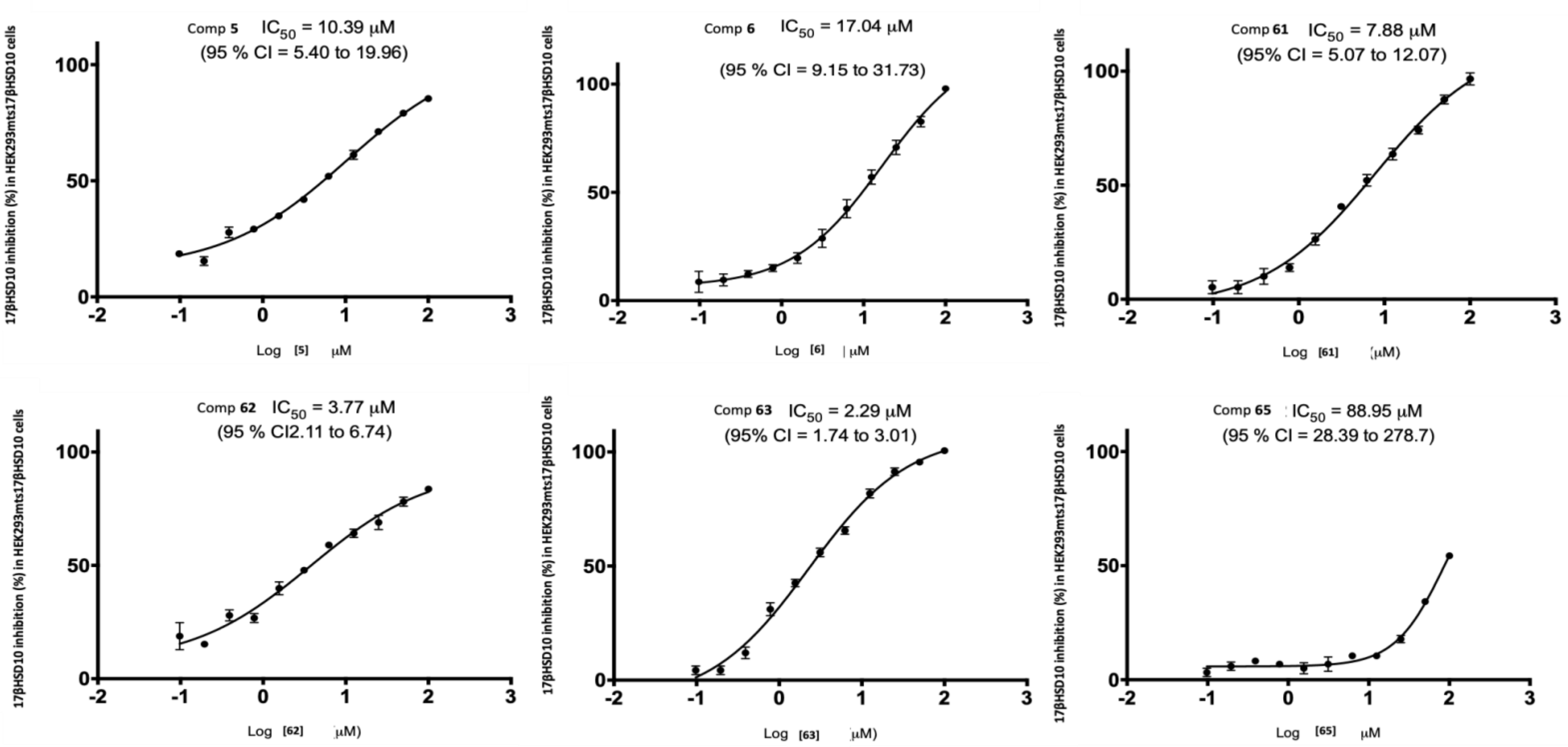

Figure [S5]: Cellular IC_{50} Graphs for compounds **5**, **6**, **61**, **62**, **63**, **64**, **65**, **66**, **67** and **78**.

3. References:

[1] L. Hroch, O. Benek, P. Guest, L. Aitken, O. Soukup, J. Janockova, K. Musil, V. Dohnal, R. Dolezal, K. Kuca, T.K. Smith, F. Gunn-Moore, K. Musilek, Design, synthesis and in vitro evaluation of benzothiazole-based ureas as potential ABAD/17β-HSD10 modulators for Alzheimer's disease treatment, Bioorg. Med. Chem. Lett. 26 (2016) 3675–3678. doi:10.1016/j.bmcl.2016.05.087.

[2] D.W. Engers, J.R. Field, U. Le, Y. Zhou, J.D. Bolinger, R. Zamorano, A.L. Blobaum, C.K. Jones, S. Jadhav, C.D. Weaver, P.J. Conn, C.W. Lindsley, C.M. Niswender, C.R. Hopkins, Discovery, Synthesis, and Structure−Activity Relationship Development of a Series of N -(4-Acetamido)phenylpicolinamides as Positive Allosteric Modulators of Metabotropic Glutamate Receptor 4 (mGlu 4) with CNS Exposure in Rats, J. Med. Chem. 54 (2011) 1106–1110. doi:10.1021/jm101271s.

[3] D. Ménard, I. Niculescu-Duvaz, H.P. Dijkstra, D. Niculescu-Duvaz, B.M.J.M. Suijkerbuijk, A. Zambon, A. Nourry, E. Roman, L. Davies, H.A. Manne, F. Friedlos, R. Kirk, S. Whittaker, A. Gill, R.D. Taylor, R. Marais, C.J. Springer, Novel potent BRAF inhibitors: Toward 1 nM compounds through optimization of the central phenyl ring, J. Med. Chem. 52 (2009) 3881–3891. doi:10.1021/jm900242c.

[4] A. V. Nizovtsev, A. Scheurer, B. Kosog, F.W. Heinemann, K. Meyer, Synthesis of differently substituted tacn-based ligands: Towards the control of solubility and electronic and steric properties of uranium coordination complexes, Eur. J. Inorg. Chem. 2013 (2013) 2538–2548. doi:10.1002/ejic.201201549.

[5] Y. Jin, Z.Y. Zhou, W. Tian, Q. Yu, Y.Q. Long, 4′-Alkoxyl substitution enhancing the anti-mitotic effect of 5-(3′,4′,5′-substituted)anilino-4-hydroxy-8-nitroquinazolines as a novel class of anti-microtubule agents, Bioorg. Med. Chem. Lett. 16 (2006) 5864–5869. doi:10.1016/j.bmcl.2006.08.058.

[6] P.Y. Bounaud, S. Hopkins, E. Jefferson, M. Wilson, M. Wong, T. Kanouni, Pyrazolothiazole Protein Kinase Modulators, U.S. Patent 11/560,460, 2007.

[7] S.S. Reddy, V.K. Rao, B.S. Krishna, C.S. Reddy, P.V. Rao, C.N. Raju, Synthesis, Antimicrobial, and Antioxidant Activity of New α-Aminophosphonates, Phosphorus Sulfur Silicon Relat. Elem. 186 (2011) 1411–1421. doi:10.1080/10426507.2010.514682.

[8] V.N. Telvekar, H.M. Bachhav, V.K. Bairwa, A Novel System for the Synthesis of 2-Aminobenzthiazoles using Sodium Dichloroiodate, Synlett. 23 (2012) 2219–2222. doi:10.1055/s-0032-1317080.
